# Extra-Axial Cavernous Angioma: A Case Report and Review of the Literature

**DOI:** 10.3390/neurolint16010010

**Published:** 2024-01-12

**Authors:** Shakiba Hassanzadeh, Linlin Gao, Anthony M. Alvarado, Paul J. Camarata, Nelli S. Lakis, Mohammad Haeri

**Affiliations:** 1 Department of Pathology and Laboratory Medicine, East Carolina University, Greenville, NC 27834, USA; 2 Department of Pathology and Laboratory Medicine, University of Kansas Medical Center, Kansas City, KS 66160, USA; 3 Department of Neurosurgery, University of Kansas Medical Center, Kansas City, KS 66160, USA

**Keywords:** extra-axial cavernous angioma, cavernous angioma, cavernous hemangioma, cavernoma, intracranial

## Abstract

Cavernous angiomas (CAs) are benign vascular malformations predominantly seen in the brain parenchyma and therefore referred to as intra-axial. Extra-axial dural-based cavernous angiomas, on the other hand, are rare vascular lesions found outside of the brain parenchyma. They occur in the middle fossa and may be easily misdiagnosed as meningiomas due to their extra-axial location. In addition, CAs that are located outside the middle fossa, such as in the convexity, have a better prognosis since they are more surgically accessible. Surgical resection is the main treatment of choice in CAs. However, other options, such as embolization and radiotherapy, may also be considered therapeutic choices or additive treatment options. The pathogenesis of CA and the involvement of other factors (genetics or environmental factors) are still unknown and require further investigation. We are presenting a young man who presented for evaluation of seizure-like events without any family history of neurologic conditions. The physical examination was unremarkable except for a slightly antalgic gait. Imaging studies showed an extra-axial left tentorial mass suggestive of a meningioma, hemangiopericytoma, or other extra-axial lesions. The lesion was resected where its vascular nature was mentioned initially, and the histology proved the diagnosis of cavernous angioma. Here we give an overview of the known pathogenesis, causes, clinical features, and diagnostic and therapeutic options in CA. Better knowledge about CA, its causes, clinical features, and treatment options would help clinicians in early diagnosis and patient management.

## 1. Introduction

Cavernous angiomas (CAs) are benign vascular malformations that have emerged from enlarged sinusoidal vessels. They appear in clusters and are lined with a thin endothelial wall with no tissue between them. In addition, they do not have elastic lamina or smooth muscles but are occasionally ossified/calcified. Cavernous angiomas are non-neoplastic vascular abnormalities [1,2,3]. Luschka was the first to describe CA, which was incidentally found in a suicidal patient in 1853 [4,5]. The terms “cavernous angioma”, “cavernous hemangioma”, “cavernous malformation”, or “cavernoma” have been used in the literature for these lesions [2,3,6,7]. CAs may occur in the brain parenchyma, spinal cord, and extra-axial regions [1]. Most of the intracranial CAs are intraparenchymal (intra-axial CAs), but extra-axial CAs are rare. In addition, dural-based CAs or CAs in other atypical sites may be misdiagnosed as meningiomas or neoplasms [1,8,9]. We aimed to give an overview of the known pathogenesis, causes, clinical features, and diagnostic and therapeutic options in CA.

## 2. Case Presentation

**Figure 1:**
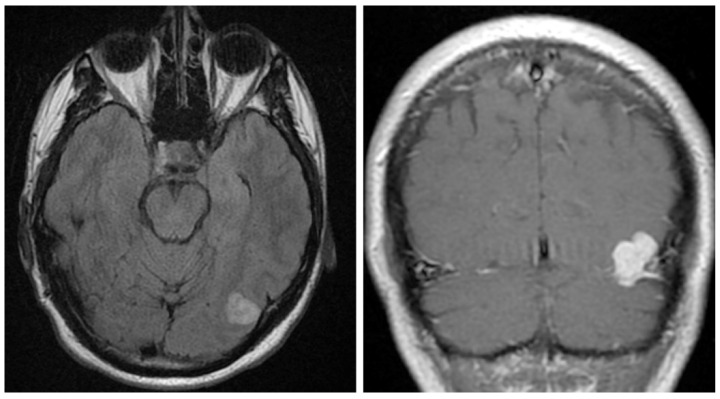
Imaging studies. Head MRI with and without contrast identified a 2.2 cm lobulated, mildly T2/FLAIR hyperintense (**left**), T1 isointense, homogeneously enhancing mass along the left tentorium (**right**).

A 40-year-old gentleman presented for evaluation of several episodes of brief unresponsiveness, lasting approximately 30 seconds. The patient had a history of four seizure-like episodes after events like having his blood drawn, being in a hot tub, and hitting his knee. No family history of neurologic conditions was noted. The physical examination was unremarkable except for a slightly antalgic gait due to a recent ankle sprain. An unenhanced head CT demonstrated a 1.7 cm hyperdense nodule extending up from the left tentorium. Head MRI with and without contrast identified a 2.2 cm lobulated, mildly T2/Fluid attenuated inversion recovery (FLAIR) hyperintense, T1 isointense, homogeneously enhancing mass along the left tentorium (Figure 1). The lesion also showed small, punctate areas of hypo-intensity on susceptibility-weighted imaging. The differential diagnosis included meningioma and hemangiopericytoma (aka. Solitary fibrous tumor), given the FLAIR hyperintensity and hyperenhancement. The patient underwent a left craniotomy with a resection of the mass. Intraoperatively, the mass arose from the tentorium and extended posteriorly to the wall of the transverse sinus. The lesion was vascular in nature and resembled a cavernous angioma. The lesion was submitted for histopathological examination.

**Figure 2:**
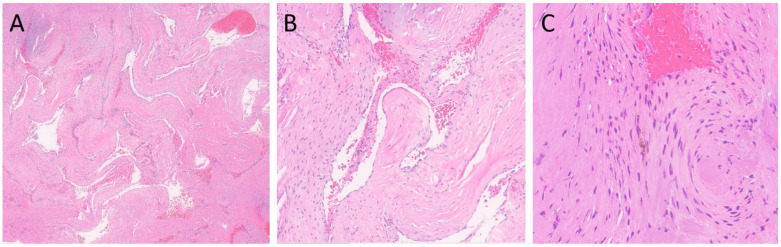
H&E-stained sections of the lesion. (**A**) Clusters of closely juxtaposed vascular channels with thick, hyalinized walls (H&E, 20× magnification). (**B**) The vessels were lined with flattened endothelial cells, and the vascular spaces were filled with blood and thrombi (H&E, 100× magnification). (**C**) Rare hemosiderin-laden macrophages were present (H&E, 200× magnification).

**Figure 3:**
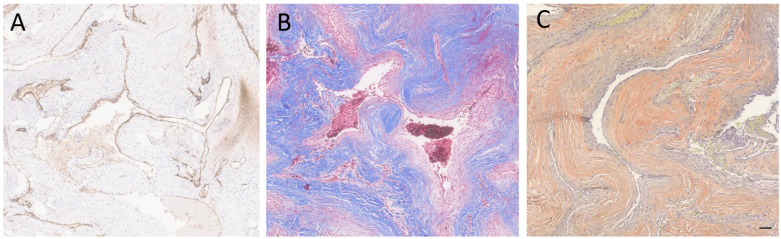
Special and immunostained sections of the lesion. (**A**) The immunohistochemical stain for CD34 highlighted the endothelial cells. (**B**) Immunohistochemical stain for trichrome highlighted the hyalinized vessel walls. (**C**) A special stain for VVG showed the absence of internal elastic lamina. Scale bar: 100 µm.

Standard laboratory procedures were performed to prepare Hematoxylin and Eosin (H&E)-stained glass slides [10], followed by performing standard immunohistochemistry per the manufacturer’s recommendations. A microscopic examination of the lesion revealed clusters of closely juxtaposed vascular channels with thick, hyalinized walls. No intervening neural or glial tissue was identified. The vessels were lined with flattened to cuboidal endothelial cells, and some of the vascular spaces were filled with blood and thrombi. Rare hemosiderin-laden macrophages were noted (Figure 2). Immunohistochemical stain for CD34 (Roche Diagnostics, Rotkreuz, Switzerland—Cat # 790-2927) highlighted the endothelial cells, and trichrome stain highlighted the hyalinized vessel walls. Verhoeff Van Gieson (VVG) (Poly Scientific, Bay Shore, NY, USA—Cat#: k059-16oz) elastic stain was negative for internal elastic lamina, which is a feature of arteries (Figure 3). The absence of an internal elastic lamina ruled out the diagnosis of arteriovenous malformation. To differentiate from meningioma and hemangiopericytoma, histochemical stains for Somatostatin receptor 2 (SSTR2) (performed at Mayo Clinic Laboratory, Rochester, MN, USA), Epithelial membrane antigen (EMA) (Cell Marque^TM^ Tissue Diagnostics, Rocklin, CA, USA), and signal transducer and activator of transcription 6 (STAT6) (Cell Marque^TM^ Tissue Diagnostics) were performed, which were all negative. The lesion was also negative for S100 (Roche Diagnostics, Switzerland-Cat# 790-2914) and SOX-10 (Cell Marque^TM^ Tissue Diagnostics—Cat # 383R-18); which made the diagnosis of neuroma or schwannoma unlikely. In summary, the histological and immunophenotypic findings were compatible with a cavernous angioma (cavernoma). The postoperative course was uneventful, and the patient was discharged on the third postoperative day. At the 1-month follow-up, the patient was doing well. The patient has had no further episodes of unresponsiveness.

We performed a literature search through the National Library of Medicine, the National Center for Biotechnology Information (https://pubmed.ncbi.nlm.nih.gov/, accessed on 1 November 2023), and Google Scholar (https://scholar.google.com/, accessed on 1 November 2023) for similar case reports and included the most relevant cases in this review.

## 3. Discussion

CAs are benign vascular malformations that account for 3–13% of intracranial vascular malformations and mostly occur in the brain parenchyma (intra-axial CAs). Extra-axial CAs occur in about 0.4% to 2% of intracranial vascular malformations [8]. CAs (or cavernomas) occur in both genders with no specific gender predominance and usually occur in the second to fifth decades of life [1]. However, there have been reports of a female predominance in extra-axial CAs [11]. In addition, CAs in newborns are rare, but there have been some reported cases diagnosed with prenatal ultrasound evaluation [12,13,14]. Gross et al. have reported that intracranial cavernous malformations are located in the supra-tentorial hemisphere (lobar) (66%), brainstem (18%), basal ganglia, thalamus, corpus callosum, or insula (deep supra-tentorial) (9%), and cerebellum (6%) [15]. Lewis et al. (1994) have described two types of extra-axial dural-based CAs [16]. The first type, which consists of the majority of extra-axial CAs, occurs in the dura of the middle cranial fossa. They mostly originate from the sellar and parasellar regions, especially the cavernous sinus [17]. The other type originates from the convexity, cerebral flax, cerebellar flax, tentorium, posterior fossa, anterior fossa (the floor), intrapetrous facial nerve, fifth nerve, eighth nerve, or skull base [2,18,19].

The exact underlying pathogenesis of dural-based cavernomas is still unclear [1]. Since CAs have been reported in the neonate population, it has been suggested that they are probably caused by abnormal vascular development of the embryos. In addition, it has been reported that genetics might have a possible role in the development of CAs [20], although CAs may also develop spontaneously [21,22]. For example, intra-parenchymal CA has been associated with the following cerebral cavernous malformation (CCM) genes: *CCM-1*, *CCM-2,* and *CCM-3*. Most of the patients with CCM and a genetic form have an autosomal dominant pattern and are mostly loss-of-function mutations of the *CCM* genes. A ‘second hit’ in an existing embryonal nonfunctioning *CCM* gene causes complete loss of function, leading to the proliferation of the endothelial cells [23,24]. It has been suggested that similar mechanisms may be involved in extra-parenchymal CMs, and consequently, these lesions may be more likely endothelial cell tumors than vascular malformations [24]. CAs may gradually enlarge due to some factors such as thrombosis, engorgement, feeding from adjacent vessels, hemorrhage, hormones, growth, changes in flow, sepsis, trauma, or after surgery [3,25]. It has also been reported that dural-based cavernous malformations (CMs) may change in morphology, increase in size, and develop angiogenesis during pregnancy. These changes have been associated with female hormones (such as estrogen and progesterone) and vascular growth factors (such as vascular endothelial growth factor), which are released during pregnancy [26,27]. For example, in 2021, Ishii et al. reported a case of dural-based CM at the temporal convexity in a pregnant patient that presented with hemorrhage [28]. On the other hand, there have also been reports of dural-based CMs without hemorrhage in pregnancy. Furthermore, the exact association between cavernous hemangioma and meningioma is still unclear. However, there have been suggestions that since both tumors may have a ventricular localization, there may be a collision of the two different tumors due to the migration of tumor cells through the cerebrospinal fluid. In addition, associations between CA and radiation or head traumas have also been suggested [29,30,31].

The clinical presentations of CAs vary and depend on the location and size of the tumor [32]. The majority of CAs are asymptomatic and may be incidentally found on autopsy or imaging [5,33,34]. Dural-based cavernomas have a wide range of non-specific clinical presentations, including seizure (37%), hemorrhage (36%), headache (23%), and neurological deficits (22%). In addition, focal neurological deficits such as sensorimotor deficits, dysphasia, and cranial nerve impairments/palsies are observed in 35–50% of the patients when the motor cortex, speech region, basal ganglia, or brainstem are involved [1,9,17,35]. Dural-based CMs that are located outside of the middle fossa are rarely associated with intracranial hemorrhage [28]. However, there have been reports of subdural hematoma in dural-based CMs located at the dural convexity [36,37].

A definitive diagnosis before surgery is important to plan the surgical technique. However, the radiological and clinical findings in CAs may not be able to distinguish extra-axial CAs from other lesions. For example, there have been reports of misdiagnosing extra-axial CA with meningiomas or neoplasms. Consequently, neurosurgeons may have to alter the excision technique during surgery and change the subsequent treatment plan. Therefore, considering extra-axial CAs as a differential diagnosis during surgery seems necessary [1]. The main differential diagnoses of extra-axial CAs include intra-axial/intraparenchymal cavernomas and meningiomas. Other differential diagnoses include cavernous malformations, metastatic neoplasms, solitary fibrous tumors/hemangiopericytoma, neuromas, high-flow vascular malformations (fistulas and arteriovenous angiomas), schwannomas, lymphomas, and sarcoidosis [1,38,39,40].

Following radiological improvements, the diagnosis of extra-axial CAs is increasing. However, confirmation with pathological evaluation is necessary, as a definite diagnosis of most CAs is only established by histological examination [1,39]. On macroscopic evaluation, CAs appear as purple, mulberry-appearing masses that are multi-loculated. These lesions have multiple irregularly arranged sinusoidal vascular channels that are separated by fibrous strands and fibrous connective tissue stroma. However, there are no neural tissues in between them (2). In addition, they are reported to originate from capillaries. On microscopic evaluation, there is a rim of a single layer of endothelial cells at the vessel wall that do not contain muscle or elastic tissue. In addition, there may be calcifications, ossifications, intravascular thrombosis, and hyalinization [5,39]. However, there are no tight junctions [24]. There have been varying reports on the size and volume range of CAs. For example, a size range of 1 mm to 75 mm, even up to 140 mm (mean size of 14.2 mm), have been reported. In general, sizes of 50–60 mm or more are considered giant CA [25].

Dural-based CAs appear different from a CA that originates from the brain parenchyma on computed tomography (CT) scan and magnetic resonance imaging (MRI) images but resemble a meningioma [41]. Since most dural-based lesions in adults are meningiomas, dural cavernomas with dural attachments may appear as meningiomas [1,42]. Differentiation between meningiomas and dural-based cavernomas with radiological evaluations is usually difficult before surgery. On contrast-enhanced CT scans, meningiomas appear as homogenous or heterogeneous lesions. They usually have a dural tail sign on MRI (with gadolinium). However, angiography of meningiomas is usually negative but may occasionally show a slight vascular redness [18,36]. On CT scans, CAs are well-circumscribed, hyperdense lesions with minimal enhancement after infusion of an iodinated contrast agent, which makes them indistinguishable from a meningioma. They do not appear with adjacent edema or a significant mass effect, and the presence of a dural tail is very rare. Some CAs may contain low-density areas that have been associated with prior thrombosis or cystic degeneration [5,39,43]. Furthermore, dural cavernomas do not cause brain edema, which may be a helpful characteristic of meningioma [24]. On a CT scan, intraparenchymal cavernomas appear as contrast-enhanced and hyperdense lesions [1,44]. Parenchymal cavernomas appear with increased size and recurrent bleeding. The increase in size in these lesions may be due to capillary hyperplasia or thrombosis in the vascular spaces [1,45]. However, bleeding and subarachnoid hemorrhage are very rare in dural-based cavernomas [42,46].

On MRI, intraparenchymal cavernomas appear as iso/hypointense and mixed/hyperintense lesions on T1-weighted and T2-weighted images, respectively [44]. In addition, a peripheral hypointense ring (hemosiderin) surrounding the parenchyma is usually observed on MRI [47]. However, peripheral hemosiderin rings are not commonly seen in dural-based cavernomas [1,44]. Dural-based CAs have a higher intensity and hyperintense signal on T2-weighted imaging compared to meningioma, which is typical for dural CAs [45]. High intensity in T2-weighted imaging has relatively high specificity. MRI and angiography findings can usually establish a preoperative diagnosis [48]. CA in the cavernous sinus of the middle cranial fossa has an intermediate signal intensity in Tl-weighted imaging and has homogeneous hyperintensity in T2-weighted imaging. In addition, following administration of intravenous (IV) gadolinium diethylenetriaminepentaacetic acid (Gd-DTPA), significant and homogeneous enhancement occurs (similar to a meningioma) [41]. Although most dural-based CAs have MRI results similar to those of a meningioma, some may show features resembling those of an intra-axial CA. For example, Vogler et al. reported a case of a dural-based CA in the occiput with MRI presentations similar to an intra-axial CA, including heterogeneous signal intensity in both short and long TR and hemosiderin deposition [5,41].

An angiogram may be used to exclude a tumor [45]. The result of catheter angiography is usually negative in these patients, but a slight vascular blush may be observed in some cases [5]. In addition, the angiogram may show vessel dislocations, widened veins, slight neovascularity, and a flecked tumor blush [33,39]. The sunburst of vessels that radiate outwards from the central vascular pedicle, which is typically seen in meningiomas, has not been reported in CAs [49]. There have been some reports that Thallium^201^ single-photon emission CT (SPECT) shows low uptake within CA lesions, while in meningioma or malignant tumors it shows high uptake. The high uptake in tumors is because of the increased viability or blood flow of the tumors [49,50]. However, there is controversy over Thallium201 SPECT helping diagnose cavernous sinus hemangioma due to the contradictory results that range from none to mild and significant uptake [34].

The treatment of choice for symptomatic dural-based cavernoma is surgery. Adjuvant therapy would be unnecessary following total surgical removal [1,28]. Uzunoglu et al. recommended considering surgery for extra-axial lesions, especially when the radiological findings are suggestive of a meningioma. Therefore, histopathological confirmation of dural hemangioma would be possible [1]. However, the site of these lesions and MRI results are important in the decision-making process of surgery [1,28]. For example, surgical resection of the CAs involving the cavernous sinus is usually difficult and may be associated with intraoperative blood loss. This may also lead to an incomplete surgical excision. Therefore, embolization, radiotherapy, or radiosurgery may be required in such cases. However, surgical removal of the dural CAs outside the cavernous sinus is usually complete without the need for other therapies [45]. Furthermore, a frozen section may be requested for the diagnosis of CA during surgery [51].

Embolization, radiotherapy, or radiosurgery may be considered in patients with deeper extra-axial lesions [1]. For example, surgical resection could be difficult due to the vascularity of extra-axial CAs, hemorrhage during surgery, cranial nerve involvement, or carotid artery involvement. Therefore, radiotherapy has been suggested as a preoperative or additive treatment option for these patients [39]. A surgical biopsy followed by radiotherapy before surgery has been suggested in CA. However, CA has unpredictable and varying radiosensitivity [52,53]. Furthermore, embolization before surgery could be helpful in cases with high vascularity [3,39]. Among the methods used for sinus CA that decrease the tumor size or vascularity and, in turn, reduce hemorrhage during surgical resection, radiosurgery has been preferred. This is because radiosurgery has shown good clinical outcomes, lower morbidity, and a lower risk of bleeding [54,55].

Furthermore, although CAs are benign lesions, some CAs may grow in size. Different factors have been reported to be involved in the growth of dural CA, including endocrine factors, capillary budding, ectasia, thrombosis of vascular spaces, or hemorrhage, which may be involved in the growth of dural cavernous angioma [37,56]. In addition, dural-based cavernomas located at the cavernous sinus and middle cranial fossa are more vascular and clinically aggressive compared to cavernomas in the convexity or infra-tentorium [41]. Extra-axial intracranial CAs that arise from the cavernous sinus tend to bleed massively during surgery, and, in turn, surgical resection is usually not successful. However, the location of extra-axial intracranial CAs that originate from the convexity is easier for surgical removal, and the bleeding during surgery is much less. Therefore, they have a better prognosis compared to those CAs that originate from the cavernous sinus [43]. Overall, most patients with CA have a good clinical outcome following surgery [25].

## 4. Conclusions

Extra-axial dural-based CA is a rare intracranial vascular lesion. It usually occurs in the middle fossa. However, those CAs that are located outside the middle fossa, such as in the convexity, have a better prognosis since they are more surgically accessible. Extra-axial dural-based CA may be misdiagnosed as meningioma. Surgical resection remains the main treatment choice in CAs. However, embolization and radiotherapy may also be considered therapeutic choices or additive treatment options. The exact pathogenesis of sporadic CA and the involvement of other factors, such as genetics or environmental factors, are still unknown and require further investigation. Better knowledge about CA, its pathogenesis, causes, clinical features, and treatment options would help clinicians promptly diagnose and manage patients with CA and, in turn, increase their quality of life and clinical outcome.

The following tables summarize the findings of reported cases in the literature based on their locations, as follows. Table 1: Extra-axial cavernous angioma of the cavernous sinus and sellar, parasellar, and intrasellar regions. Table 2. Dural-based cavernous hemangiomas in convexities. Table 3: Cavernous angioma in the falx cerebri. Table 4. Extra-axial cavernoma of the tentorium. Table 5. Extra-axial cavernoma of the cerebellopontine angle (CPA). Table 6. Extra-axial cavernous angioma of the cerebellar falx.

## Figures and Tables

**Table 1:** Extra-axial cavernous angioma of the cavernous sinus and sellar, parasellar, and intrasellar regions.

	Article	Year	Age (Years)	Gender	Clinical Features	Location	Management	Outcome
1.	Kamrin et al. [57]	1965	46	Female	Bifrontal headache Left hemicranial pain Progressive left-sided vision loss Right-sided ptosis on the right	Sellar Right parasellar	Surgery	Death (11 days after surgery)
2.	Sansone et al. [58]	1980	72	Female	History of metastatic breast cancer Progressive transient double vision	Sellar Left parasellar	Autopsy	-
3.	Buonaguidi et al. [59]	1984	50	Male	History of cavernous hemangioma (8 years ago) Headache Weakness Cold intolerance Constipation Impotence Seizure Reduced visual acuity (on both sides) Superior bitemporal quadrantanopia Bilateral optic pallor Hypopituitarism	Sellar Suprasellar Left parasellar	Surgery	Recurrence after 8 years (reoperation)
4.	Sawamura et al. [60]	1990	45	Female	Progressive right-sided visual disturbance Reduced left-sided visual acuity Transient diplopia Left hemiparesis Limb and truncal ataxia Sensory disturbance in the lower extremities Left retrobulbar optic neuritis Diagnosed as multiple sclerosis Left-sided blurred vision		Surgery	Recovery of left homonymous hemianopsia 6th cranial nerve palsy (resolved after six months)
5.	Mitsuhashi et al. [61]	1991	45	Female	History of neurofibromatosis Progressive left-sided vision loss Headache Nausea	Sellar Left parasellar	Surgery	Transcalvarial brain herniation and death (during surgery)
6.	Chhang et al. [62]	1991	48	Male	Headache Blurred vision Reduced left-sided vision Right nasal inferior quandrantanopia Bilateral constriction of visual fields Mild temporal pallor of the optic discs	Sellar Suprasellar Right parasellar	Surgery Irradiation	Uneventful postoperative course
7.	Lombardi et al. [63]	1994	41	Female	Irregular periods Galactorrhea Hyperprolactinemia Transient diplopia	Sellar Left parasellar	Surgery	-
8.	Cobbs et al. [64]	2001	41	Male	Asymptomatic Left posterior orbital hemangioma found while working up for sinusitis	Sella (right part)	Surgery	Subarachnoid hemorrhage CSF rhinorrhea (9 days after surgery) Placement of a lumboperitoneal shunt (10 days after surgery)
9.	Biondi et al. [3] (Case 1)	2002	24	Female	Progressive 6th left cranial nerve ophthalmoplegia Left-handed face pain and hypesthesia in the 6th cranial nerve	Left cavernous sinus	Surgery	No clinical worsening
10.	Biondi et al. [3] (Case 2)	2002	-	-	Ophthalmoplegic migraine	-	Surgery	No clinical worsening
11.	Biondi et al. [3] (Case 3)	2002	-	-	Headache	-	Surgery	No clinical worsening
12.	Biondi et al. [3] (Case 4)	2002	38	Female	Hypoesthesia and paresthesia of left cranial nerves V1 and V2	Left cavernous sinus	Pre-operative embolization Biopsy Angiographic devascularization	No clinical worsening
13.	Jeon et al. [65]	2004	63	Male	Reduced visual acuity Bitemporal hemianopia Hypopituitarism Hyponatremia	Sellar Left parasellar	Surgery	Improvement of hyponatremia
14.	Chuang et al. [55]	2006	62	Female	Progressive right-sided ptosis Right-sided horizontal gaze and inferolateral deviation Anisocoric pupils Diplopia Partial oculomotor palsy	Sellar Right parasellar	Surgery	Improvement of diplopia
15.	Turan et al. [51]	2013	32	Female	Blurred vision	Sellar Suprasellar	Surgery	Transient diabetes insipidus
16.	Ma et al. [66]	2014	-	-	Headache Loss of libido Blurred vision	Sellar Left parasellar	Surgery	-
17.	Oommen et al. [2]	2017	50	Male	Left occipital headache Double vision Left 6th nerve palsy Partial left 3rd nerve palsy	Left parasellar area	Surgery	Asymptomatic
18.	Wu et al. [67]	2017	64	Female	Headache Progressive loss of vision at both sides Bitemporal visual field deviation Hyperprolactinemia	Sellar Right parasellar	Surgery	Improvement of visual acuity
19.	Das et al. [68] (Case 1)	2018	66	Female	Episodic headache Bitemporal hemianopia	Sellar Right parasellar	Surgery	-
20.	Das et al. [68] (Case 2)	2018	48	Female	Headache Galactorrhea	Sellar Left parasellar	Surgery	-
21.	Al-Sharydah et al. [69]	2018	43	Male	Headache Blurred vision Decreased libido Impotence Reduced left-sided visual acuity Bitemporal homonymous hemianopsia Bilateral 6th nerve palsy	Sellar Suprasellar	Surgery Stereotactic radiosurgery	Hypothyroidism
22.	Chibbaro et al. [70]	2018	49	Male	Progressive left eye ptosis Strabismus Diplopia Headache 3rd nerve palsy Homolateral dilated pupil reactive to light Superior temporal quadrant anopia	Sellar Left parasellar	Surgery Stereotactic radiation	Further lesion shrinkage is stable after five years of follow-up
23.	Pan et al. [71]	2020	55	Female	Intermittent dizziness	Intrasellar	Surgery	Good condition No changes in lesion volume on MRI (after a 2-year follow-up)
24.	Al-Saiari et al. [72]	2021	49	Female	Symptomatic nonfunctional pituitary macroadenoma Chronic bitemporal headache Fatigue Progressive reduction in vision Decreased visual acuity on both sides (more on the left) Bitemporal homonymous hemianopia	Sellar Suprasellar Right parasellar	Surgery Stereotactic radiosurgery	Improvement of visual acuity and field Adequate decompression of the optic chiasm and nerves

**Table 2:** Dural-based cavernous hemangiomas in convexities.

	Article	Year	Age (Years)	Gender	Clinical Features	Location	Management	Outcome
1.	Canevini et al. [13]	1963	Neonate	-	-	-	-	-
2.	Okada et al. [73]	1977	35	Male	Loss of grasp in the right hand Generalized convulsion Soft swelling in the left supraorbital area Positive Barre’s pyramidal sign in the lower right extremity	Left supraorbital area	Surgery	-
3.	Ito et al. [74]	1978	-	-	-	Parietal convexity	-	-
4.	Kunishio et al. [20]	1986	61	Female	Left blepharoptosis Diplopia Left oculomotor nerve palsy	Convexity	Surgery	-
5.	Saldana et al. [12]	1991	Neonate	Male	Fetus sonogram: Hyperechogenic mass on the surface of the left hemisphere	Mass abutting the inner table of the skull in the frontoparietal region	Surgery	Uneventful postoperative course
6.	Perry et al. [75]	1993	77	Female	Partial seizure	Left parietal convexity	Surgery	Uneventful recovery
7.	Revuelta et al. [43]	1994	66	Male	Headache Increased left intraocular pressure Decreased retinal sensitivity in the superior and nasal regions	Left occipito-temporal gyrus	Surgery	Uneventful postoperative course
8.	Lewis et al. [16]	1994	36	Female	Vertigo Global Headache Bilateral horizontal nystagmus Visual acuity: 20/30 (both sides) Left facial weakness	Right parietal convexity	Surgery	Right occipital headaches (eleven months after surgery) Surgical removal of the right occipital dural CA resolved the right occipital headaches
9.	Vogler et al. [5]	1995	35	Male	Generalized seizures Headache Left visual blurring	Right parietal occipital	Surgery	-
10.	Suzuki et al. [37]	1996	78	Female	Surgery for transient ischemic attacks (1989) Headache Vomiting Loss of consciousness Subdural hematoma	Frontal convexity	Surgery	Successful surgery, but without complete recovery of consciousness
11.	McKechnie et al. [76]	1998	47	Female	Episodic visual disturbance Left-sided flashes of light Left-sided visual blurring Partial left upper homonymous quadrantanopia Asymmetry of the optic nerves with the appearance of the left optic disc (mild glaucomatous change with normal intraocular pressure)	Convexity of the right occipital lobe (lateral to the falx)	Surgery	Transient mild decrease in the visual acuity of the left eye (resolved by discharge)
12.	Hyodo et al. [77]	2000	77	Male	Consciousness disturbance	Right parieto-occipital convexity	Surgery	-
13.	Shen et al. [41]	2000	18	Female	Left temporal pain Left facial pain	Left parietal lobe	Surgery	Unremarkable
14.	Puca et al. [39]	2004	32	Female	Progressive left exophthalmos Frontal headache Vertigo Left 5th cranial nerve paresthesia Hypesthesia in the first two divisions of the left trigeminal nerve 6th nerve palsy	Parietal convexity		Uneventful postoperative course Removal of a painful subcutaneous left parietal hemangioma (13 months later)
15.	Hwang et al. [22]	2009	61	Male	Vertigo Headache History of minor head injury (age 12) History of right frontal lytic lesion (age 45) Post-operative intracranial hemorrhage	Right frontal convexity	Surgery	-
16.	Joshi et al. [78]	2009	15	Male	Headache (left parietal region)	Left parieto-occipital convexity	Surgery	Asymptomatic
17.	Sakakibara [79]	2010	59	Male	Neurological deficits	Left parieto-occipital convexity	Surgery	Resolution of numbness
18.	Yonezawa et al. [48]	2014	78	Female	Headache	Convexity	Surgery	-
19.	Kashlan et al. [7]	2014	56	Male	Episodic right-sided visual flashes History of the left occipital mass (seven years ago)	Left occipital convexity	Surgery	Uneventful postoperative course No recurrence
20.	Di Vitantonio H et al. [80]	2015	30	Female	Progressive left frontal headache	Left frontal	Surgery	Asymptomatic
21.	Wang et al. [45]	2015	37	Female	Sensory disturbance of the right limbs	Left parietal		No recurrence
22.	Pelluru et al. [81]	2018	26	Male	Seizure Papilledema	Left temporoparietal	Surgery	Asymptomatic
23.	Bhide et al. [82]	2018	22	Female	Seizure Headache	Right frontal convexity	Surgery	Uneventful postoperative course No recurrence
24.	Li et al. [83]	2018	33	Male	Right frontal (forehead) subcutaneous lump History of a car accident (6 years ago)	Falx cerebri Right frontal convexity dura	Surgery	Stable residual lesions at a three-month follow-up
25.	Dubovoy et al. [31]	2018	63	Female	Headaches (right frontal region and right orbit)	Supratentorial, frontal convexity	Surgery	Uneventful postoperative course No recurrence at 1-year follow-up
26.	Biteich et al. [32]	2019	67	Male	History of aortic aneurysm surgery Jerking movements on the right side of his body Distorted writing Right-sided facial movements	Left frontoparietal	Surgery	Asymptomatic No recurrence
27.	Ishii et al. [28]	2021	29	Female	Headache at 38 weeks of pregnancy	Left temporal	Surgery	No recurrence

**Table 3:** Cavernous angioma in the falx cerebri.

	Article	Year	Age (Years)	Gender	Clinical Features	Location	Management	Outcome
1.	Fracasso et al. [84]	1947	47	Female	-	-	-	-
2.	Kaga et al. [85]	1991	62	Female	Intermittent headache Dizziness No neurological deficit	Beneath the falx cerebri	Surgery	-
3.	Biondi et al. [3]	2002	63	Female	Worsened headache Recent dizziness and falls	Anterior third of the flax	Surgery	No clinical worsening
4.	Dorner et al. [34]	2005	37	Male	Paranoid schizophrenia	Right frontal	Surgery	Discharged for further psychiatric management
5.	Kim et al. [49]	2006	22	Female	Generalized tonic-clonic seizure No focal neurologic deficit	Right frontal	Surgery	-
6.	Simonin et al. [6]	2018	61	Female	Increasing behavioral disturbances Worsening neuropsychological symptoms Gait instability Memory loss	Frontobasal lesion	Surgery	Persistent symptoms of a depressed mood Diminished capacity to concentrate Improved gait instability
7.	Uzunoglu et al. [1]	2019	58	Male	Headache Dizziness No neurological deficits	Posterior interhemispheric fissure near the posterior part of the corpus callosum splenium	Surgery	-

**Table 4:** Extra-axial cavernoma of the tentorium.

	Article	Year	Age (Years)	Gender	Clinical Features	Location	Management	Outcome
1.	McCormic et al. [86]	1966	52	Male	Schizophrenia Death due to coronary artery thrombosis	Right leaf of the tentorium cerebelli	Autopsy	-
2.	McCormic et al. [86]	1966	54	Male	Death due to suppurative cholangitis, liver necrosis, and uremia	Right leaf of the tentorium	Autopsy	-
3.	Huber [87]	1968	28	Female	Headache	-	Surgery	-
4.	Moritake et al. [14]	1985	Fetus/Neonate	Female	Fetus: Ventromegaly Craniomegaly Posterior fossa mass	Right tentorium cerebelli	Surgery	Uneventful postoperative course Normal development
5.	Matsumoto et al. [88]	1988	61	Female	Left auditory disturbance Left hemifacial spasm	Left tentorium cerebelli	Surgery	-
6.	Quattrocchi et al. [21]	1989	63	Male	Frontal headaches Mild dementia	Tentorium cerebelli	Surgery	Uneventful postoperative course
7.	Lee et al. [89]	1998	53	Male	Homonymous hemianopsia		Surgery	
8.	Van Lindert et al. [25]	2006	36	Female	Headache Nausea Vomiting Dizziness Difficulties in writing Unsteady gait Distracted and indifferent onneurological examination Ataxia Positive Romberg test	Temporoparietal lesion in the right hemisphere with transtentorial extension in the right cerebellar hemisphere	Surgery	Uncomplicated recovery No recurrence
9.	Mori et al. [8]	2009	15	Male	Headache Left-sided scintillation	Right cerebellar tentorium with extension to the supratentorial and infratentorial spaces	Surgery	Transient left homonymous hemianopia
10.	Bhatia et al. [24]	2011	60	Male	Shaking of left hand with activity Mild truncal ataxia Gait disturbance	Tentorial mass, with its bulk primarily in the posterior fossa	Surgery	Resolution of neurologic deficits
11.	Yoshimura et al. [90]	2014	15	Female	Transient left scintillation (2 years ago) Diplopia Bilateral papilledema Left homonymous scotoma Headache	Right occipital and suboccipital regions, both the supra- and infratentorial spaces	Preoperative endovascular embolization Surgery	Symptoms recovered except for the left homonymous scotoma Delayed healing of the wound

**Table 5:** Extra-axial cavernoma of the cerebellopontine angle (CPA).

	Article	Year	Age (Years)	Gender	Clinical Features	Location	Management	Outcome
1.	Iplikcioglu et al. [91]	1986	30	Female	Left-sided hearing loss Headache Left facial palsy Bilateral papilledema Hypesthesia of the V1-V2 areas	Left CPA	Surgery	Uneventful postoperative course Left peripheral facial palsy and total health loss of the left ear (at 6-year follow-up)
2.	Goel et al. [92]	1993	60	Male	Episodic ataxia of left-sided limbs	Lateral part of the left cerebellar hemisphere	Surgery	Uneventful postoperative course
3.	Brunoni et al. [93]	1996	60	Male	Right facial numbness Tinnitus Hearing loss Vertigo Imbalance Right 5th to 8th cranial nerve deficits Right-sided cerebellar signs	Left CPA	Surgery	-
4.	Kim et al. [94]	1997	32	Male	Left facial hypesthesia Asymmetrical sensorineural hearing loss Facial paresis	CPA without internal auditory canal involvement	Surgery	Resolution of facial paresis
5.	Ferrante et al. [95]	1998	24	Female	Right anacusia Nausea and vomiting Right-sided vestibular impairment Positive Romberg test	Right CPA	Surgery	Regression of mild cranial nerve VII deficit (within 10 days) Persistent right anacusia
6.	Vajramani et al. [96]	1998	46	Male	Tinnitus of right ear Headache Clumsiness of upper and lower limb (right side) Difficulty in walking Slurred speech Bilateral horizontal gaze-dependent nystagmus Right-sided sensorineural hearing loss Cerebellar signs	Right CPA	Surgery	Post-operative evaluation showed residual tumor Total excision with surgical re-exploration: ◦ Improved hearing and tinnitus ◦ Persistent sensorineural hearing loss
7.	Benkonakli et al. [97] (Case 1)	2002	19	Male	Dizziness Nausea and vomiting Right-sided sensorineural hearing loss Right facial numbness Right-sided hypesthesia (V1/V2) Truncal ataxia Mild right-sided facial palsy	Involvement of the seventh-eighth nerve complex	Surgery	Persistent facial hypoesthesia
8.	Benkonakli et al. [97] (Case 2)	2002	25	Male	Right-sided facial numbness Right-sided hearing loss Hypesthesia in V1/V2 areas Mild right-sided hearing loss	Right CPA	Surgery	Good condition
9.	Deshmukh et al. [68] (Case 1)	2003	76	Male	Progressive left-sided hearing loss Dysphagia Facial droop	Left CN VII/CN VIII	Surgery	Improved facial paresis Stable hearing
10.	Deshmukh et al. [98] (Case 2)	2003	53	Male	Progressive left-sided sensorineural hearing loss Facial droop Facial paresis (House-Brackmann Grade IV)	Left CPA, Left CN VII/CN VIII	Surgery	Improved facial paresis Stable hearing
11.	Stevenson et al. [99]	2005	57	Male	Right-sided sensorineural hearing loss Tinnitus Facial numbness Gait imbalance Facial hypesthesia High-frequency hearing loss	Right CPA	Surgery	Resolution of symptoms Restoration of hearing
12.	Albanese et al. [100]	2009	48	Male	Dizziness Vomiting Tinnitus Voice change Hoarseness Gait instability	Lower third of the right CPA cistern	Surgery	Significant improvement in voice tone
13.	Sasani et al. [19]	2010	16	Female	Headache	Right cerebellopontine angle	Surgery	Uneventful postoperative course No residual lesion
14.	Engh et al. [101]	2010	16	Female	Left-sided hearing loss Near-total unilateral sensorineural deafness Gait instability House-Brackmann Grade III facial paresis	Left cerebellopontine angle	Surgery	Normal facial nerve function Permanent hearing loss
15.	Huang et al. [102]	2011	50	Male	Right-sided sensorineural hearing loss Vertigo Facial numbness Unsteady gait Ataxia Right-sided facial hypoesthesia Right-sided high-frequency hearing loss	Cerebellopontine angle	Surgery	Resolution of symptoms
16.	Otani et al. [103]	2012	74	Female	Hearing disturbance Tinnitus Vertigo Ataxia	Cerebellopontine angle cistern	Surgery	Hearing could not be recovered
17.	Wu et al. [104]	2012	36	Male	Left-sided facial palsy Facial numbness Homolateral hearing loss House-Brackmann Grade V facial paresis (left side) Left-sided sensorineural hearing loss Left-sided hypesthesia	Left cerebellopontine angle cistern	Surgery	Resolution of facial numbness Improvement of facial paresis Slight alleviation of hearing disturbances
18.	Ghanta et al. [40]	2013	50	Male	Dysarthria Dysphagia Unsteady gait Left-sided lower cranial nerve palsy Left-sided cerebellar signs	Left cerebellopontine angle	Surgery	Significant recovery of the lower cranial nerve palsy
19.	Tarabay et al. [17]	2019	44	Female	Bilateral tinnitus Vertigo Nausea and vomiting Mild sensorineural hearing loss Nystagmus	Right cerebellopontine angle	Surgery	Subtotal resection of the lesion, with a small residual part Recovery from vertigo and gait instability Unchanged hypoacousia

**Table 6:** Extra-axial cavernous angioma of the cerebellar falx.

	Article	Year	Age (Years)	Gender	Clinical Features	Location	Management	Outcome
1.	Ito et al. [105]	2009	47	Male	Incidental finding No neurological symptoms	Posterior cranial fossa arising attached to the cerebellar falx	Surgery	Unremarkable postoperative course
2.	Melone et al. [42]	2010	58	Male	An episode of mental confusion	Posterior cranial fossa arising from the cerebellar falx	Surgery	Uneventful postoperative course

## Data Availability

Any data relevant to this manuscript are available by request according to regulations set forth by the KUMC research institution.
